# Supplementary material for: circNUDT21 promotes bladder cancer progression by modulating the miR-16-1-3p/MDM2/p53 axis
Source: Mol Ther Nucleic Acids. 2021 Sep 6;26:625–36. doi: 10.1016/j.omtn.2021.08.032 (PMC8517098; doi:10.1016/j.omtn.2021.08.032)
Supplement: Document S1. Table S1 [file mmc1.pdf]

OMTN, Volume *26*

Supplemental information

circNUDT21 promotes bladder cancer progression by modulating the miR-16-1-3p/MDM2/p53 axis

Liang Chen, Wencheng Li, Zhiqin Li, Yarong Song, Jun Zhao, Zhaohui Chen, Gallina Kazobinka, Lulu Li, Yifei Xing, and Teng Hou

Supplemental Table 1. The sequences of primers, oligonucleotides and probes used in this study.

**PCR**	CircNUDT21-divergent	Forward	AATGCACCAGGATATGGACCC
		Reverse	TGAAGCTGAACCAGAAACAACT
	CircNUDT21-convergent	Forward	CCTTGTTTGCAGTCCCTAAAAAT
		Reverse	CAACAGCTGAGGGAGACTAGAA
	GAPDH-divergent	Forward	TCCCCCACCACACTGAATCT
		Reverse	ACCACCTGGTGCTCAGTGTA
	GAPDH-convergent	Forward	TTTGGCTACAGCAACAGGGTG
		Reverse	TACATGGCAACTGTGAGGAGGG
	Circ_0039441-divergent	Forward	GTTTGGTTTTGAGAACTTTTCTGGT
		Reverse	GGGTCCATATCCTGGTGCAT
	Circ_0039442-divergent	Forward	GTTTGGTTTTGAGAACTTTTCTGGT
		Reverse	GGCGTAGGCACGCCG
**qRT-PCR**	CircNUDT21	Forward	AATGCACCAGGATATGGACCC
		Reverse	TGAAGCTGAACCAGAAACAACT
	NUDT21	Forward	CCCCACCTTAGGATGGTGAA
		Reverse	GGAGTTGCACCTTGGACTCA
	MDM2	Forward	ACGACAAAGAAAACGCCACA
		Reverse	GTAACTTGATATACACCAGCATCAA
	GAPDH	Forward	AAATCAAGTGGGGCGATGCTG
		Reverse	GCAGGAGGCATTGCTGATGAT
	miR-3162-5p	Forward	CGCGTTAGGGAGTAGAAGGGT
		Reverse	AGTGCAGGGTCCGAGGTATT

miR-6511b-5p	Forward	CTGCAGGCAGAAGTGGGG
	Reverse	AGTGCAGGGTCCGAGGTATT
miR-4793-5p	Forward	CGACATCCTGCTCCACAGGG
	Reverse	AGTGCAGGGTCCGAGGTATT
miR-6129	Forward	CGCGTGAGGGAGTTGGG
	Reverse	AGTGCAGGGTCCGAGGTATT
miR-4739	Forward	GGGAGGAGGAGCGGAGG
	Reverse	AGTGCAGGGTCCGAGGTATT
miR-16-1-3p	Forward	CGCGCCAGTATTAACTGTGC
	Reverse	AGTGCAGGGTCCGAGGTATT
miR-4482-5p	Forward	GCGCGAATTGAATAGTGAGCA
	Reverse	AGTGCAGGGTCCGAGGTATT
miR-8052	Forward	CGCGGGACTGTAGAGGGC
	Reverse	AGTGCAGGGTCCGAGGTATT
miR-4635	Forward	GCGCGTCTTGAAGTCAGAAC
	Reverse	AGTGCAGGGTCCGAGGTATT
miR-6874-5p	Forward	CGATGGAGCTGGAACCAGA
	Reverse	AGTGCAGGGTCCGAGGTATT
miR-19b-1-5p	Forward	GCGAGTTTTGCAGGTTTGCA
	Reverse	AGTGCAGGGTCCGAGGTATT
miR-3130-5p	Forward	GCGACCCAGTCTCCGGTG
	Reverse	AGTGCAGGGTCCGAGGTATT
miR-6834-5p	Forward	GCGGTGAGGGACTGGGAT

	Reverse	AGTGCAGGGTCCGAGGTATT
miR-6794-5p	Forward	CGCAGGGGGACTGGGG
	Reverse	AGTGCAGGGTCCGAGGTATT
miR-6760-5p	Forward	CGCAGGGAGAAGGTGGAAG
	Reverse	AGTGCAGGGTCCGAGGTATT
miR-509-5p	Forward	CGCGTACTGCAGACAGTGG
	Reverse	AGTGCAGGGTCCGAGGTATT
miR-3911	Forward	GCGTGTGTGGATCCTGGAG
	Reverse	AGTGCAGGGTCCGAGGTATT
miR-4686	Forward	GCGTATCTGCTGGGCTTTCT
	Reverse	AGTGCAGGGTCCGAGGTATT
miR-6825-5p	Forward	CGTGGGGAGGTGTGGAGT
	Reverse	AGTGCAGGGTCCGAGGTATT
miR-5047	Forward	CGTTGCAGCTGCGGTTG
	Reverse	AGTGCAGGGTCCGAGGTATT
miR-6829-5p	Forward	GCGTGGGCTGCTGAGAA
	Reverse	AGTGCAGGGTCCGAGGTATT
miR-6127	Forward	CGCGTGAGGGAGTGGGT
	Reverse	AGTGCAGGGTCCGAGGTATT
miR-6133	Forward	CGCGTGAGGGAGGAGGT
	Reverse	AGTGCAGGGTCCGAGGTATT
miR-4510	Forward	CGCGTGAGGGAGTAGGATGT
	Reverse	AGTGCAGGGTCCGAGGTATT

	miR-22-3p	Forward	GCGAAGCTGCCAGTTGAAG
		Reverse	AGTGCAGGGTCCGAGGTATT
	miR-3199	Forward	GCGAGGGACTGCCTTAGGAG
		Reverse	AGTGCAGGGTCCGAGGTATT
	miR-1301-3p	Forward	TTGCAGCTGCCTGGGAGT
		Reverse	AGTGCAGGGTCCGAGGTATT
	U6	Forward	CTCGCTTCGGCAGCACA
		Reverse	AACGCTTCACGAATTTGCGT
**RT-PCR**	miR-3162-5p	GTCGTATCCAGTGCAGGGTCCGAGGTATTCGCACTGGATACGACCTCCCC	
	miR-6511b-5p	GTCGTATCCAGTGCAGGGTCCGAGGTATTCGCACTGGATACGACTGTCAG	
	miR-4793-5p	GTCGTATCCAGTGCAGGGTCCGAGGTATTCGCACTGGATACGACCCTCTG	
	miR-6129	GTCGTATCCAGTGCAGGGTCCGAGGTATTCGCACTGGATACGACTATACA	
	miR-4739	GTCGTATCCAGTGCAGGGTCCGAGGTATTCGCACTGGATACGACAGGGCC	
	miR-16-1-3p	GTCGTATCCAGTGCAGGGTCCGAGGTATTCGCACTGGATACGACTCAGCA	
	miR-4482-5p	GTCGTATCCAGTGCAGGGTCCGAGGTATTCGCACTGGATACGACCTGGGT	
	miR-8052	GTCGTATCCAGTGCAGGGTCCGAGGTATTCGCACTGGATACGACGCTCAT	
	miR-4635	GTCGTATCCAGTGCAGGGTCCGAGGTATTCGCACTGGATACGACTTGCGG	
	miR-6874-5p	GTCGTATCCAGTGCAGGGTCCGAGGTATTCGCACTGGATACGACGCCTGA	
	miR-19b-1-5p	GTCGTATCCAGTGCAGGGTCCGAGGTATTCGCACTGGATACGACGCTGGA	

miR-3130-5p	GTCGTATCCAGTGCAGGGTCCGAGGTATTCGCACTGGATACGACAGGCTG
miR-6834-5p	GTCGTATCCAGTGCAGGGTCCGAGGTATTCGCACTGGATACGACCCACAA
miR-6794-5p	GTCGTATCCAGTGCAGGGTCCGAGGTATTCGCACTGGATACGACGCTCAC
miR-6760-5p	GTCGTATCCAGTGCAGGGTCCGAGGTATTCGCACTGGATACGACTCTGCA
miR-509-5p	GTCGTATCCAGTGCAGGGTCCGAGGTATTCGCACTGGATACGACTGATTG
miR-3911	GTCGTATCCAGTGCAGGGTCCGAGGTATTCGCACTGGATACGACTGCCTC
miR-4686	GTCGTATCCAGTGCAGGGTCCGAGGTATTCGCACTGGATACGACAACACC
miR-6825-5p	GTCGTATCCAGTGCAGGGTCCGAGGTATTCGCACTGGATACGACATGCTG
miR-5047	GTCGTATCCAGTGCAGGGTCCGAGGTATTCGCACTGGATACGACACCTTA
miR-6829-5p	GTCGTATCCAGTGCAGGGTCCGAGGTATTCGCACTGGATACGACTGCCCC
miR-6127	GTCGTATCCAGTGCAGGGTCCGAGGTATTCGCACTGGATACGACCCTCCC
miR-6133	GTCGTATCCAGTGCAGGGTCCGAGGTATTCGCACTGGATACGACTACCCA
miR-4510	GTCGTATCCAGTGCAGGGTCCGAGGTATTCGCACTGGATACGACAACCAT
miR-22-3p	GTCGTATCCAGTGCAGGGTCCGAGGTATTCGCACTGGATACGACACAGTT
miR-3199	GTCGTATCCAGTGCAGGGTCCGAGGTATTCGCACTGGATACGACAACTTT
miR-1301-3p	GTCGTATCCAGTGCAGGGTCCGAGGTATTCGCACTGGATACGACGAAGTC
U6	AACGCTTCACGAATTTGCGT

**RNA pull-down**	CircNUDT21-probe	TCCTGACGACCCAGTATCTGCTCAACAGCTGAGG-Biotin
	Oligo probe	CCTCAGCTGTTGAGCAGATACTGGGTCGTCAGGA-Biotin
	Bio-miR-16-1-3p	CCAGUAUUAACUGUGCUGCUGA-Biotin
	Bio-miR-16-1-3p-mut	AACUGCGUAAAGGUGAUAUGAC-Biotin
