# Supplementary material for: circNUDT21 promotes bladder cancer progression by modulating the miR-16-1-3p/MDM2/p53 axis
Source: Mol Ther Nucleic Acids. 2021 Sep 6;26:625–36. doi: 10.1016/j.omtn.2021.08.032 (PMC8517098; doi:10.1016/j.omtn.2021.08.032)
Supplement: Document S2. Article plus Supplemental Information [file mmc2.pdf]

Molecular Therapy
Nucleic Acids
Original Article

circNUDT21 promotes bladder cancer progression by modulating the miR-16-1-3p/MDM2/p53 axis

Liang Chen,[1,5] Wencheng Li,[1,5] Zhiqin Li,[2,5] Yarong Song,[1] Jun Zhao,[1] Zhaohui Chen,[1] Gallina Kazobinka,[1,3] Lulu Li,[4] Yifei Xing,[1] and Teng Hou[1]

[1]Department of Urology, Union Hospital, Tongji Medical College, Huazhong University of Science and Technology, Wuhan, HB 430022, China; [2]Department of Pharmacy, Tongji Hospital, Tongji Medical College, Huazhong University of Science and Technology, Wuhan 430030, China; [3]Urology Unit, La Nouvelle Polyclinique Centrale de Bujumbura, Bujumbura 378, Burundi; [4]Reproductive Medicine Center, Union Hospital, Tongji Medical College, Huazhong University of Science and Technology, Wuhan 430022, China

Bladder cancer (BC) is a common genitourinary malignancy. This study investigated the regulatory effects of an exonic circRNA, circNUDT21, in the progression of BC. The circNUDT21 level was overexpressed in BC tissues and cell lines as compared to normal controls. Overexpression and silencing of circNUDT21 promoted and inhibited, respectively, the proliferative and invasive abilities of BC cells. Mechanistical analysis showed that circNUDT21 acted as a miR-16-1-3p sponge and that MDM2 was a potential downstream target of miR-16-1-3p. We further verified that overexpression of circNUDT21 was associated with elevated MDM2 and reduced p53 expression. CircNUDT21 promoted BC progression by acting as a sponge of miR-16-1-3p to activate the miR-16-1-3p/MDM2/p53 axis. These findings suggest that circNUDT21 functions as an oncogenic circRNA and may be a potential therapy target for BC.

INTRODUCTION

Bladder cancer (BC) is one of the most aggressive malignant tumors in the world, with >400,000 new cases and 165,000 deaths each year.[1] Approximately 30% of patients with BC were found to have muscle-invasive disease at the time of diagnosis, and the prognosis of these patients was poor, with a 5-year survival rate of <60%.[2] In addition, relapse and metastasis always occur in BC patients, which is the major obstacle to conventional therapies.[3] Therefore, elucidation of the mechanism of BC progression at the molecular level is critical for the development of effective treatment strategies.

Circular RNAs (circRNAs) are a type of single-stranded RNAs with a covalently closed continuous circular structure formed by reverse splicing of pre-mRNA.[4] Essentially, circRNAs are resistant to the digestion of exonucleases or RNase R, due to the absence of 5′ or 3′ polarities.[5] Growing evidence has shown that circRNAs may contribute to the regulation of various biological processes such as transcriptional regulation, protein translation, and immune regulation.[6] Recently, many circRNAs have been shown to be involved in the tumorigenesis and development of human cancers.[7] For example, circRNA hsa_circ_002577 has been shown to accelerate endometrial cancer progression by acting as a miR-625-5p sponge, upregulating IGF1R and activating the PI3K/Akt pathway.[8] CircTNPO3, also known as hsa_circ_0001741, has been demonstrated to contribute to the paclitaxel resistance of ovarian cancer cells at least partly by upregulating NEK2 expression by sponging miR-1299.[9] A novel protein, HER2-103, encoded by circ-HER2, acted as a promising biomarker to predict the efficacy of pertuzumab for the treatment of triple-negative breast cancer.[10]

In this study, we investigated the functional consequences of an exonic circRNA generated from the Nudix Hydrolase 21 (NUDT21) gene, called circNUDT21. NUDT21 is a conserved splicing factor and a member of the core-machinery proteins responsible for the cleavage and polyadenylation of pre-mRNAs.[11] In our previous report, we identified NUDT21 as a tumor suppressor in BC progression.[12] Here, we found that this NUDT21-derived circRNA was significantly upregulated in BC tissues and cells. Functionally, the silencing of circNUDT21 inhibited cell proliferation, migration, and invasion *in vitro* and impeded tumor growth *in vivo*. Mechanistically, circNUDT21 could sponge for miR-16-1-3p to relieve its suppression on the MDM2 proto-oncogene (MDM2), and further inhibit p53 activity. These results provide new insight into the MDM2-p53 pathway and open a new perspective for developing novel therapeutic strategies for BC. Collectively, our data demonstrate that circNUDT21 acts as an oncogenic circRNA in BC progression through the miR-16-1-3p/MDM2/p53 axis, and

Received 15 October 2020; accepted 31 August 2021;
https://doi.org/10.1016/j.omtn.2021.08.032.

[5]These authors contributed equally

Correspondence: Dr. Yifei Xing, Department of Urology, Union Hospital, Tongji Medical College, Huazhong University of Science and Technology, Wuhan, HB 430022, China.
E-mail: yfxing@hust.edu.cn
Correspondence: Dr. Teng Hou, Department of Urology, Union Hospital, Tongji Medical College, Huazhong University of Science and Technology, Wuhan, HB 430022, China
E-mail: h1aiyan@hust.edu.cn

This is an open access article under the CC BY license (http://creativecommons.org/licenses/by/4.0/).

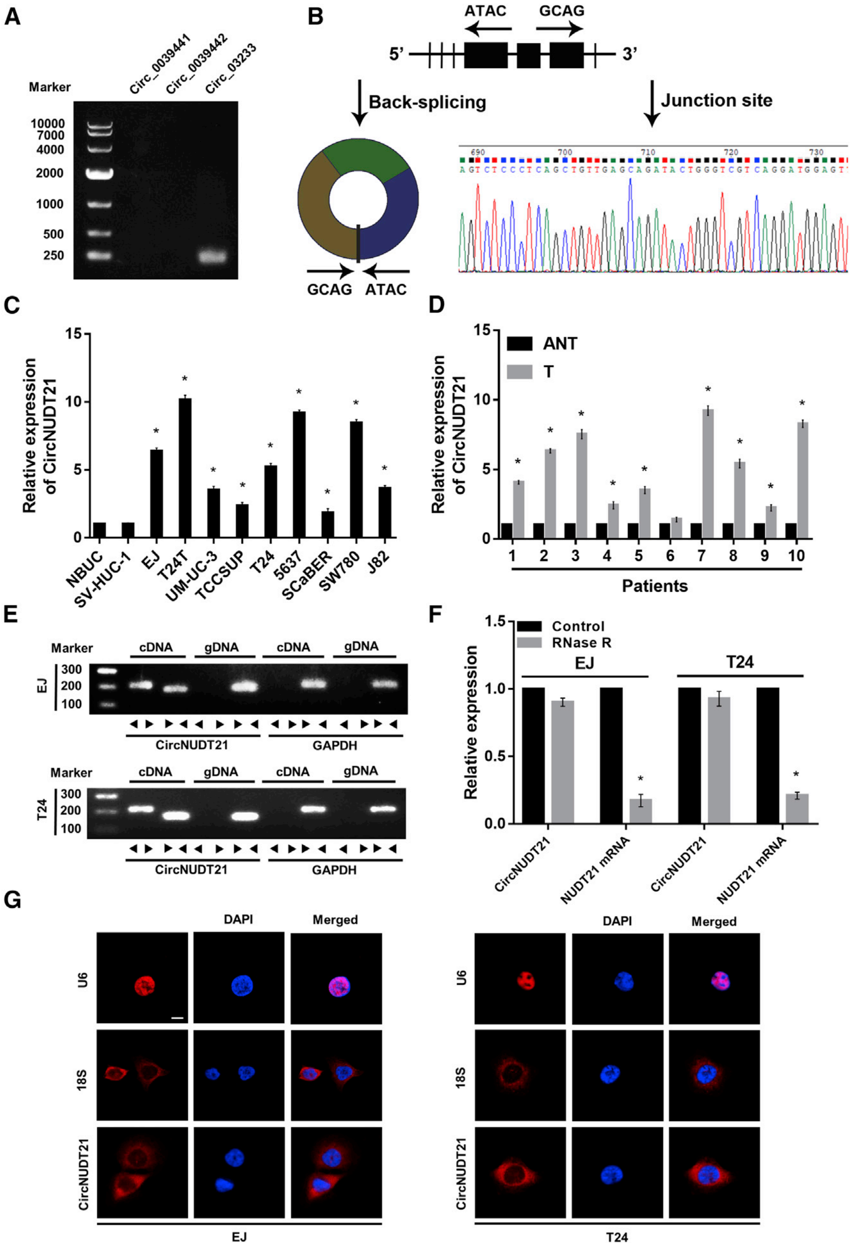

Figure 1. Identification of circNUDT21 in bladder cancer

(A) The presence or absence of circRNAs derived from NUDT21 checked by PCR with divergent primers. (B) Schematic illustration showing the structure of circNUDT21, which was verified by Sanger sequencing. (C and D) The expression levels of circNUDT21 in bladder cancer cell lines and tissues. (E) PCR assays with divergent and convergent primers indicating the existence of circNUDT21 in EJ and T24 cell lines. GAPDH was used as a linear control. (F) The expression levels of circNUDT21 and the corresponding linear product in BC cells after the treatment of RNase R. (G) RNA-FISH assays detecting the localization of circNUDT21 in BC cell lines. Scale bar, 10 μm. Data are represented as means ± SDs of 3 independent experiments. *$p < 0.05$.

may serve as a promising diagnostic marker and therapeutic target for BC.

RESULTS

circNUDT21 is upregulated in BC cells and tissues

The circRNA sequencing databases, including CircInteractome (https://circinteractome.nia.nih.gov/), circBase (http://www.circbase.org/), and circRNADb (http://reprod.njmu.edu.cn/cgi-bin/circrnadb/circRNADb.php), revealed that three potential circRNAs (circ_0039441, circ_0039442, and circ_03233) are derived from NUDT21. We performed PCR with divergent primers, and the products were analyzed by agarose gel electrophoresis assay. The results showed that only circ_03233 (CircNUDT21) was amplified in the cDNA of BC cell lines (Figure 1A). Validated by Sanger sequencing, circNUDT21 is composed of exons 4, 5, and 6 of NUDT21 (281 nt), and therefore belongs to exonic circRNAs (EcRNA) (Figure 1B). Using qRT-PCR, we showed that the expression level of circNUDT21 was markedly upregulated in BC cell lines and tissues in comparison with that in corresponding normal controls (Figures 1C and 1D). To further validate the circular construction of circNUDT21, we designed convergent and divergent primers to amplify linear and circular products of NUDT21 in cDNA and genomic DNA (gDNA). Notably, circNUDT21 was amplified by divergent primers in cDNA, while no amplification product was observed in gDNA. The linear product of NUDT21, however, was amplified in both cDNA and gDNA (Figure 1E). The RNase R treatment assay showed that circNUDT21 was resistant to RNase R, while the level of NUDT21 mRNA was significantly reduced after the treatment of RNase R (Figure 1F). The RNA-fluorescence in situ hybridization (FISH) assay revealed that circNUDT21 was mainly localized in the cytoplasm (Figure 1G).

circNUDT21 promotes the proliferation, migration, and invasion of BC cells *in vitro*

To investigate the biological role of circNUDT21 in BC cells, we constructed the circNUDT21-overexpression (CircNUDT21) and circNUDT21 knockdown (CircNUDT21-Sh#1 and CircNUDT21-Sh#2) BC cell lines (Figures 2A and 2B). The gain- or loss-of-function assays

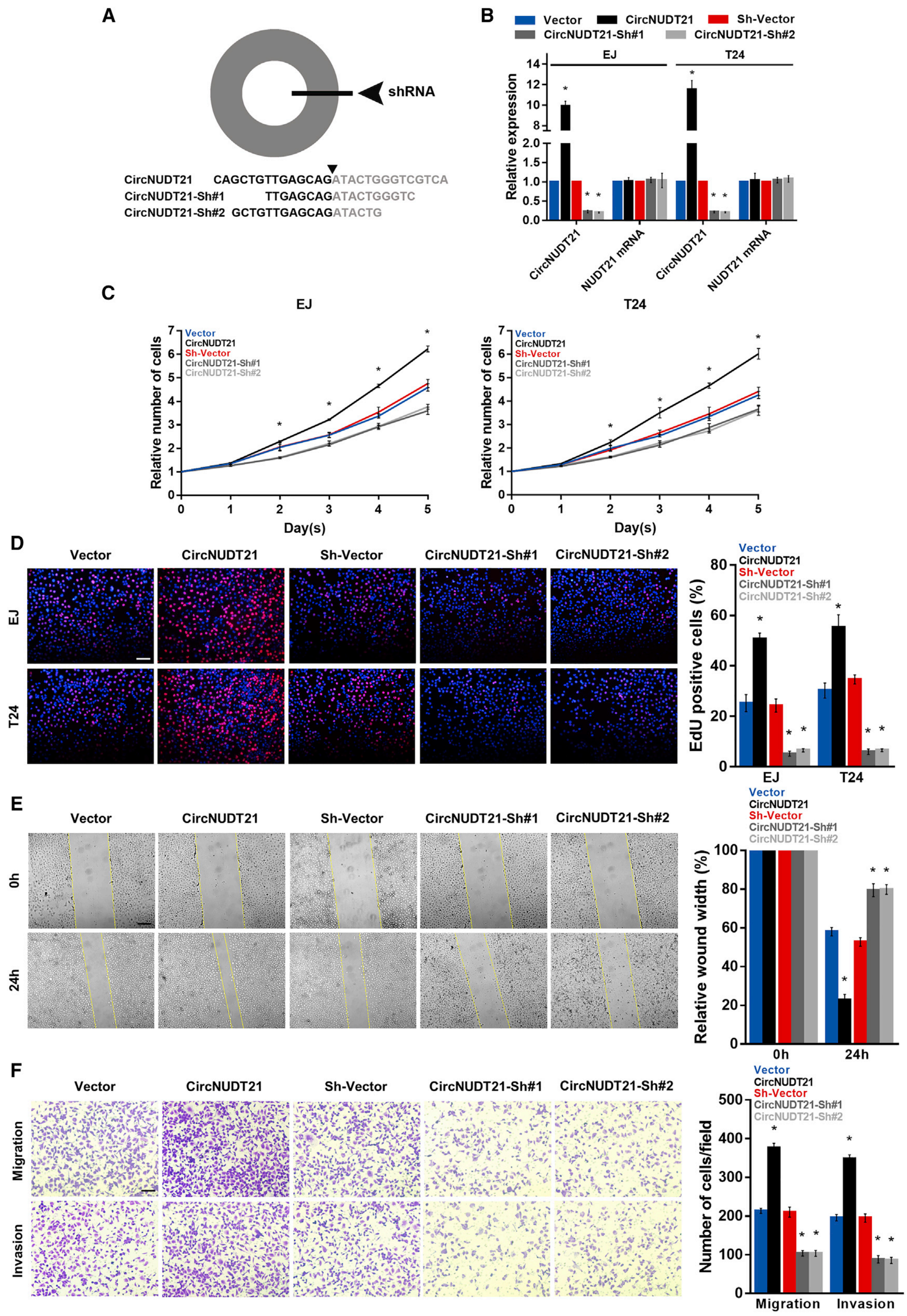

(legend on next page)

demonstrated that the ectopic expression of circNUDT21 effectively augmented, while silencing of circNUDT21 inhibited, the proliferation of BC cells (Figures 2C and 2D). Moreover, circNUDT21-transfected cells exhibited significantly increased abilities to migrate and invade (Figures 2E and 2F). These results suggested that circNUDT21 promotes the proliferation, migration, and invasion of BC cells.

circNUDT21 enhances BC growth *in vivo*

We then established a xenograft tumor model using BALB/C-nu mice to explore the impact of circNUDT21 on BC growth *in vivo*. In accordance with the *in vitro* assays, the *in vivo* study showed that tumors with overexpressing circNUDT21 were larger and heavier than those in the control group, and the opposite situation was observed in tumors with low-expressing circNUDT21 (Figures 3A–3D). Immunohistochemistry staining showed a higher expression of Ki67 in the group overexpressing circNUDT21 than in the control group (Figure 3E). These results indicated that circNUDT21 contributes to BC tumorigenicity *in vivo*.

circNUDT21 interacts with miR-16-1-3p in BC cells

Besides functioning as a miRNA sponge, circRNAs in the cytoplasm could also be translated into peptides. We explored the coding potential of circNUDT21 using the circRNADb database, but failed to find any open reading frame (ORF) in the genome of circNUDT21, indicating that the possibility of circNUDT21 encoding proteins is very low. Therefore, we next explored whether circNUDT21 could act as a miRNA sponge. To perform a pull-down assay, we designed and synthesized the specific biotinylated circNUDT21 probe (Figure 4A) and verified its efficiency and specificity. The results showed that circNUDT21 could be specifically enriched by the circNUDT21 probe (Figures 4B and 4C). By using bioinformatical tools miRanda and RNAhybrid, 27 potential miRNAs were predicted as potential targets of circNUDT21. The pull-down assay showed that only miR-16-1-3p was abundantly pulled down in both EJ and T24 cells by circNUDT21 (Figures 4D and 4E). There were three predicted binding sites between circNUDT21 and miR-16-1-3p. Among them, binding site 1 is located in the fusion region, while binding sites 2 and 3 are distal to the fusion site (Figure 4F). If binding site 1 is the binding region between circNUDT21 and miR-16-1-3p, miR-16-1-3p would not be precipitated by the circNUDT21 probe as a result of competition. Thus, we assume that binding sites 2 and 3 are potential binding regions. In addition, the abundance of circNUDT21 captured by the wild-type miR-16-1-3p probe was far more than that captured by the mutant miR-16-1-3p probe (Figure 4G). The RNA-FISH assay showed that miR-16-1-3p and circNUDT21 were co-located in the cytoplasm (Figure 4H). These results demonstrated that circNUDT21 acts as a miRNA sponge of miR-16-1-3p in BC cells.

The effect of circNUDT21 could be remedied by miR-16-1-3p

To assess whether circNUDT21 and miR-16-1-3p can co-regulate the biological function of BC cells, we enforced the expression of circNUDT21 and miR-16-1-3p simultaneously in EJ and T24 cells. The cell counting kit-8 (CCK-8), 5-ethynyl-2′-deoxyuridine (EdU), wound healing, and transwell assays indicated that miR-16-1-3p could abolish the effects of circNUDT21 on BC cell proliferation, migration, and invasion (Figures 5A–5D).

circNUDT21 stimulates BC progression via the miR-16-1-3p/MDM2/p53 axis

In this *in silico* study using 3 bioinformatics algorithms, including miRDB (http://mirdb.org/), TargetScan (http://www.targetscan.org/vert_72/), and miRmap (https://mirmap.ezlab.org/app/), we found that MDM2, which was proved to be involved in BC progression,[13] may be a potential target of miR-16-1-3p (Figures 6A and 6B). Using the dual-luciferase reporter assay, we showed that the luciferase activity of the wild-type, rather than the mutant, MDM2-3′ UTR reporter was significantly decreased in BC cells transfected with miR-16-1-3p mimics (Figure 6C). Western blotting assay showed that miR-16-1-3p could negatively regulate the expression of MDM2 (Figure 6D). Moreover, the function of circNUDT21 in upregulating the expression of MDM2 and inhibiting the expression of p53, a downstream gene of MDM2, could be compromised by miR-16-1-3p (Figure 6E). Interestingly, there was no correlation between the expression levels of circNUDT21 and miR-16-1-3p (Figure 6F). The result may be due to that the overexpression of circNUDT21 led to the decrease of the amount of miR-16-1-3p binding to MDM2-3′ UTR, while the total amount of miR-16-1-3p is stable. We next examined the expression levels of miR-16-1-3p, MDM2, and p53 in tumor xenografts from each group. The results showed that the expression level of MDM2 was upregulated, while the p53 expression was downregulated in the circNUDT21-overexpression group. The opposite expression pattern was observed in the circNUDT21 knockdown group as well. However, there was no significant difference between the 2 groups in terms of miR-16-1-3p expression, which was consistent with the result in Figure 6F (Figure 6G). To further explore the expression correlation among them, we detected the expression of circNUDT21, MDM2, and p53 in human bladder cancer tissues. The result revealed that the expression of MDM2 was remarkably upregulated, while the expression of p53 was downregulated in tissues with high levels of circNUDT21 expression (Figure 6H). All of the findings above demonstrated that circNUDT21 promotes BC progression by modulating the miR-16-1-3p/MDM2/p53 axis.

DISCUSSION

Recent evidence suggests that circRNAs may act as important tumorigenic drivers or suppressors in various human cancers.[14,15]

Figure 2. circNUDT21 promotes the aggressiveness of bladder cancer cells *in vitro*

(A) Schematic representation of the sites of the shRNAs specific to the back-splice junction of circNUDT21. (B) The expression level of circNUDT21 in indicated cells. (C) The proliferation curves of BC cells with different treatments plotted by the CCK-8 assay. (D) Representative EdU fluorescence images of BC cells in different groups. Scale bar, 100 μm. (E) Mobility of BC cells measured using the wound healing assay. Scale bar, 200 μm. (F) Representative figures of migrating and invading cells as analyzed using the transwell assay. Scale bar, 100 μm. Data are represented as means ± SDs of 3 independent experiments. $^{*}p < 0.05$.

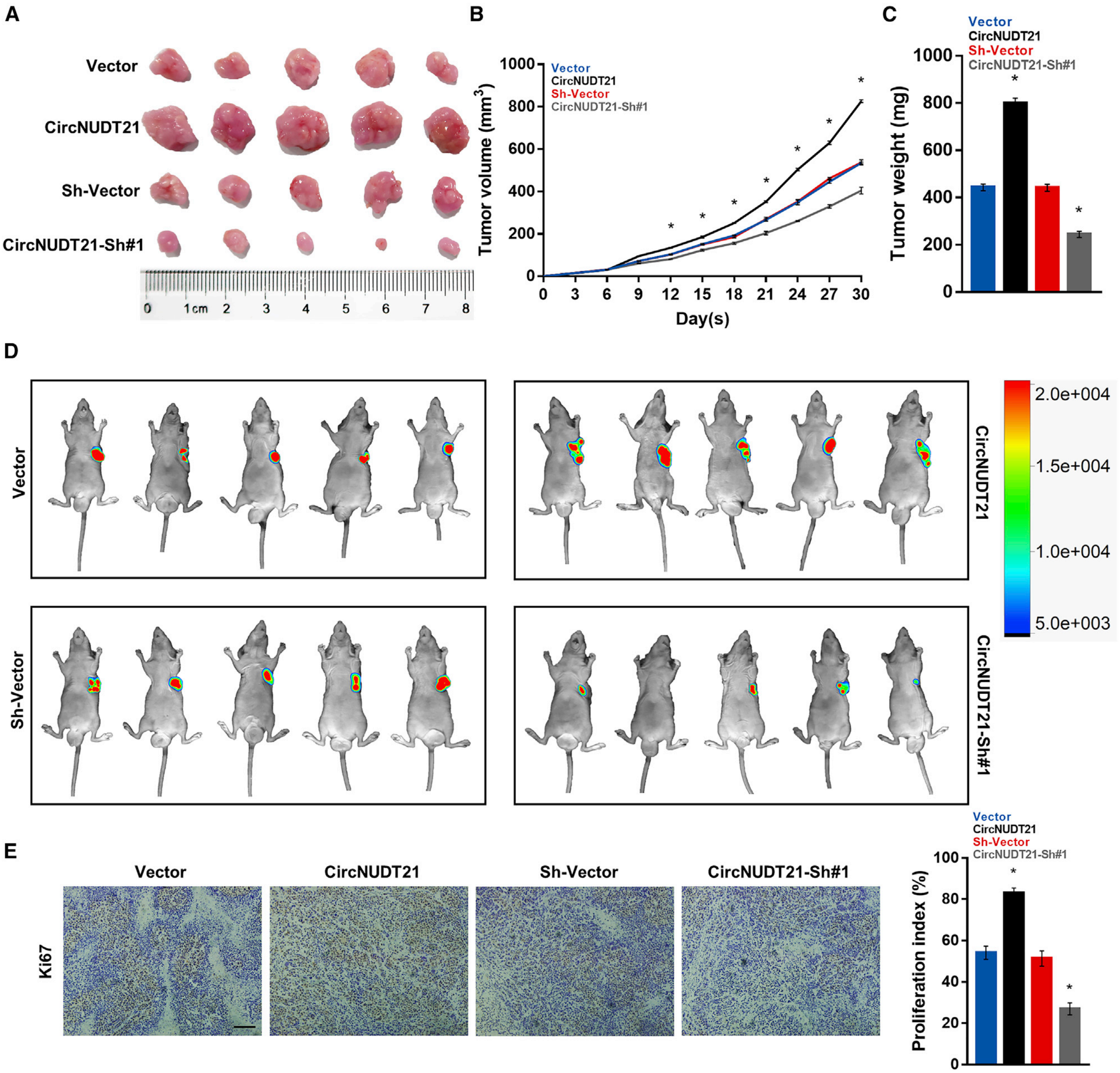

Figure 3. circNUDT21 promotes the tumorigenicity of bladder cancer *in vivo*

(A) Representative images of tumors formed in BALB/c-nu mice injected with indicated cells. (B) Tumor volumes measured on the indicated days. (C) Tumor weight in each treatment group. (D) Tumor size monitored by the *In Vivo* Optical Imaging System in indicated groups. (E) Immunohistochemical staining showing the expression of Ki67 in xenografts. Scale bar, 100 μm. Data are represented as means ± SDs of 3 independent experiments. *p < 0.05.

Moreover, some circRNAs have been proven to be related to BC progression. For instance, circ_0001944 was shown to function as a competing endogenous RNA to regulate PROK2 expression via sponging miR-548 in BC.[16] Another study showed that circRIP2 activity accelerates bladder cancer progression via inducing the epithelial-mesenchymal transition (EMT) by activating the miR-1305/Tgf-β2/smad3 pathway.[17]

In this study, we demonstrated that circNUDT21, generated from exons 4, 5, and 6 of the NUDT21 gene, may act as an oncogenic circRNA in BC through the circNUDT21/miR-16-1-3p/MDM2/p53 axis. Our results indicated that circNUDT21 expression was upregulated in BC and was associated with tumor stage and clinical prognosis. We further demonstrated that circNUDT21 promoted the proliferation, migration, and invasion *in vitro* and enhanced the tumor

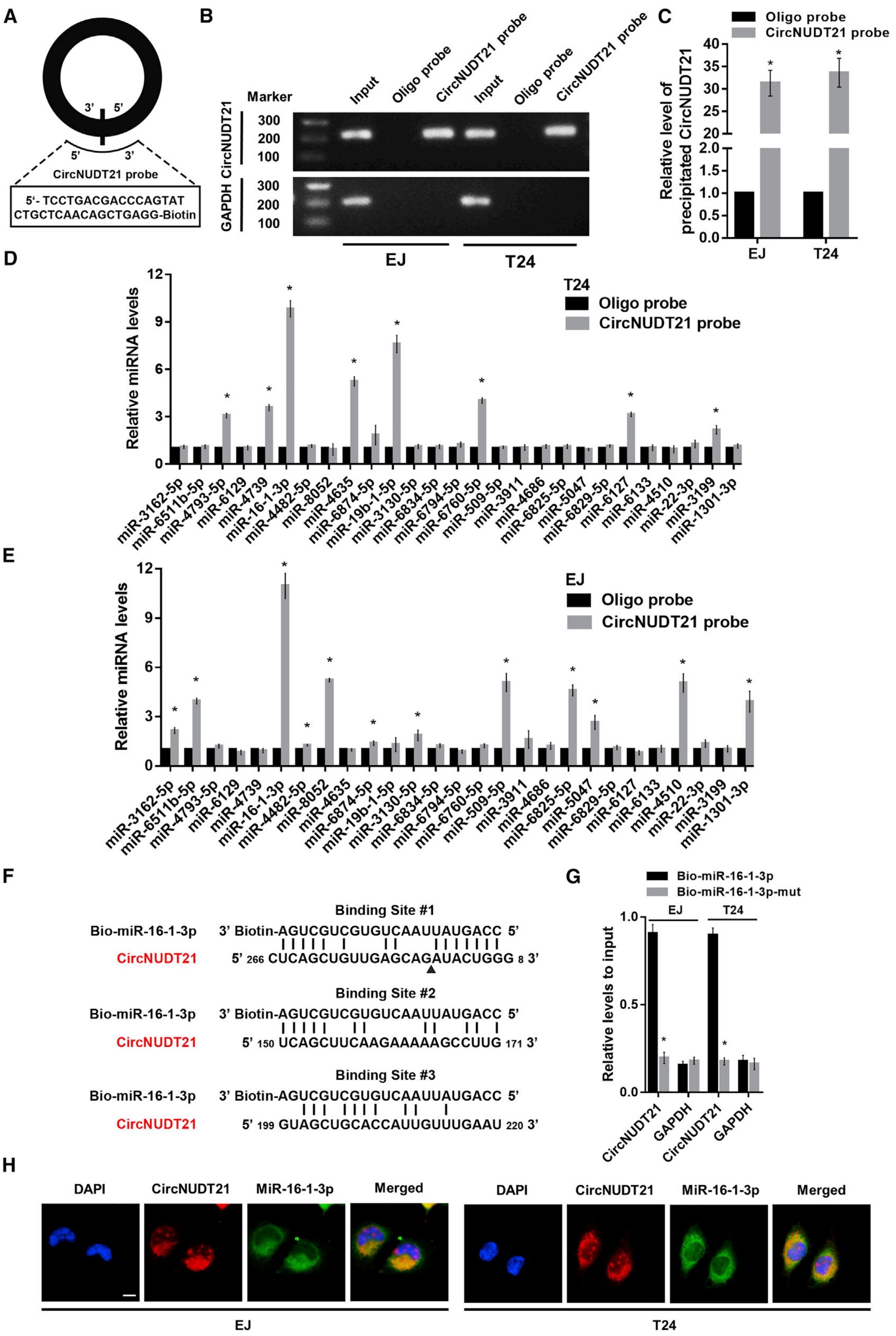

(legend on next page)

growth *in vivo*. In addition, circNUDT21 acts as a sponge to competitively bind with miR-16-1-3p and weakens its inhibitory effect on the downstream target gene MDM2, an important regulator of p53 in BC. In previous research, miR-16-1-3p was found to target TWIST1 to suppress the progression of non-small cell lung cancer and gastric cancer.[18,19] In addition, the role of miR-16-1-3p as a tumor suppressor in breast cancer through phosphoglycerate kinase 1 (PGK1) suppression was investigated.[20] In the present study, we revealed that miR-16-1-3p could directly target the 3′ UTR of MDM2 to suppress its expression, and the suppressive effect could be compromised by circNUDT21.

In our previous study, we demonstrated that NUDT21 functions as a tumor suppressor in BC and exerts its role via the modulation of alternative polyadenylation.[12] In this study, we found that circNUDT21 was highly expressed in BC and that circNUDT21 promoted the proliferative, migratory, and invasive abilities of BC cells. As circRNAs are normally described to have similar functions as their linear counterparts, our results revealed the antagonistic functions of linear and circular RNA. One possible explanation is that there is a type of intragenic regulon that can perform different biological functions by differentially regulating the expression of linear and circular components.[21] An alternative explanation is that circNUDT21 biogenesis competes with linear NUDT21 mRNA splicing, as their nascent RNAs are directed to alternative pathways that lead to linear/circular productions.[22] These findings highlighted the notion that linear and circular RNAs may have independent functions, while the underlying mechanisms need further investigation.

MDM2, as an E3 ubiquitin-protein ligase, mediates the ubiquitination of p53/TP53, leading to its degradation by the proteasome.[23] The MDM2 gene is located at chromosome 12q13.14 and was initially identified in a locus amplified on double minute chromosomes in a spontaneously transformed mouse cell line.[24] Accumulating evidence has emonstrated that MDM2 is an essential regulator of p53 by binding to a region of the TP53 transactivation domain.[25] The overexpression of MDM2 can abrogate the tumor-suppressive function of the wild-type p53 by inhibiting p53-mediated transactivation.[26] In addition, MDM2 can inhibit p53 function by targeting p53 for proteolytic degradation.[27] Moreover, MDM2 has been shown to play important roles during the tumorigenesis and progression of BC.[28,29] In this study, we further confirmed by western blotting that targeted upregulation of circNUDT21 resulted in increased MDM2 expression and reduced p53 expression. As the T24 cell line contains the mutant p53, while the EJ cell line expresses the wild-type p53, the results suggested that circNUDT21 may exert its oncogenic function through the MDM2-p53 pathway in bladder cancer, regardless of the p53 mutation status. Our finding is supported by Kim and colleagues,[30] who found that MDM2-C has E3 auto-ubiquitin ligase activity, which can promote the ubiquitination of both wild-type and mutant p53.

In conclusion, we demonstrate that circNUDT21 promotes BC progression, at least in part, through the miR-16-1-3p/MDM2/p53 axis. Our study may contribute to a better understanding of the molecular mechanism of BC development, providing a promising circRNA-targeted therapy for BC.

MATERIALS AND METHODS

Cell culture

Normal bladder urothelial cells (NBUCs) were isolated from fresh patient specimens, as described previously.[31] The uroepithelial cell line SV-HUC-1 and BC cell lines, including 5637, UM-UC-3, TCCSUP, T24, EJ, SCaBER, J82, and SW780, were purchased from the Cell Bank of the Chinese Academy of Sciences (Shanghai, China). The T24T cell line was a gift from Dr. Guosong Jiang of Wuhan Union Hospital. All of the cell lines were cultured in RPMI 1640 medium supplemented with 10% fetal bovine serum (FBS) and maintained at 37°C in a 5% CO_2 mammalian cell culture incubator.

Tissue specimens

Fresh bladder cancer tissues and matched normal tissues were obtained from patients undergoing radical cystectomy at Wuhan Union Hospital. None of the patients had received radiotherapy or chemotherapy before surgery. All of the tissues were excised carefully and placed immediately in liquid nitrogen. The study was approved by the ethics committee of the Huazhong University of Science and Technology-affiliated Union Hospital. A written informed consent form was obtained from each participant.

Reverse-transcriptase-PCR (RT-PCR) and quantitative real-time-PCR

The total RNA of cells or tissues was extracted using the Trizol kit (Invitrogen, CA, USA) according to the manufacturer's instructions, and was reversely transcribed to cDNA using the PrimeScript RT reagent kit (Takara, Japan). For mRNA and circRNA, Oligo dT Primer and Random 6 mers were used for the real-time-PCR assay, while for miRNA, a miRNA-specificity RT primer with stem-loop structure was used. Then, the StepOnePlus Real-Time PCR System (Applied Biosystems, CA, USA) was used to perform the quantitative real-time-PCR. Glyceraldehyde 3-phosphate dehydrogenase (GAPDH) and U6 were used as internal controls, and the $2^{-\Delta\Delta CT}$ method was used to evaluate the relative expression level of circRNA, miRNA, and mRNA. The sequences of primers are listed in Table S1.

Figure 4. circNUDT21 acts as a sponge for miR-16-1-3p in BC cells

(A) Schematic diagram showing the probe specifically designed for circNUDT21. (B) The specificity of circNUDT21 probe determined by the agarose gel electrophoresis. (C) The efficiency of circNUDT21 probe verified by quantitative real-time-PCR. (D) The abundances of 27 potential miRNAs pulled down by circNUDT21 in EJ cell line. (E) The abundances of 27 potential miRNAs pulled down by circNUDT21 in T24 cell line. (F) The predicted binding regions between miR-16-1-3p and circNUDT21. (G) The level of circNUDT21 enriched by the biotinylated wild-type miR-16-1-3p (Bio-miR-16-1-3p) or its mutant (Bio-miR-16-1-3p-mut). (H) RNA-FISH images showing the co-localization of circNUDT21 and miR-16-1-3p in the cytoplasm of BC cells. Scale bar, 10 μm. Data are represented as means ± SDs of 3 independent experiments. *$p < 0.05$.

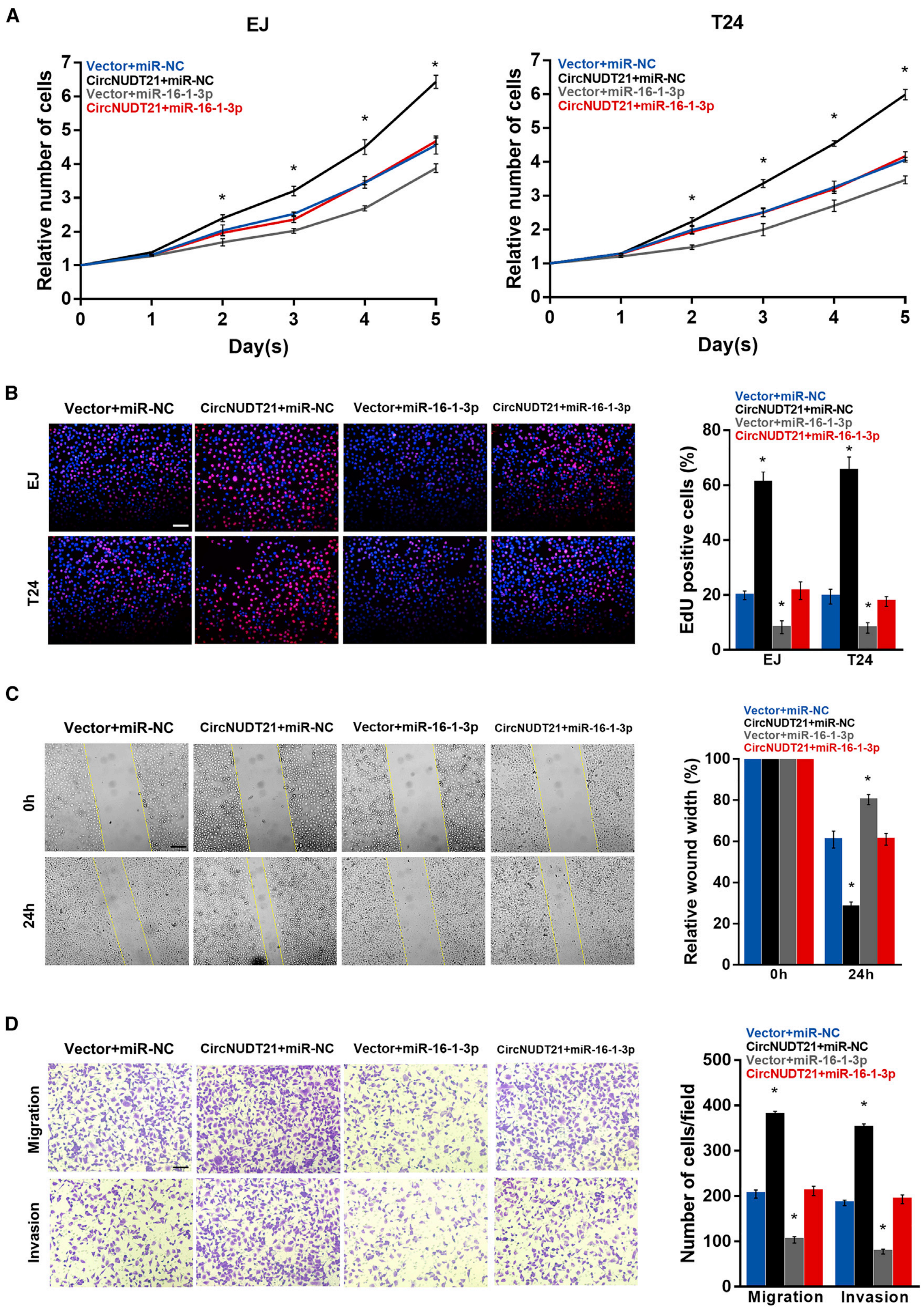

(legend on next page)

Western blotting

The RIPA Lysis and Extraction Buffer (Thermo Scientific, MA, USA) was used to extract protein from cells and tissue samples, and the Pierce BCA Protein Assay Kit (Thermo Scientific) was used to measure the protein concentration. Then proteins were electrophoresed on SDS-PAGE gels and transferred to polyvinylidene fluoride (PVDF) membranes. After that, membranes were blocked in 5% milk for 1 h at room temperature and incubated with primary antibodies overnight at 4°C, with secondary antibodies. Finally, the protein signals were detected by chemiluminescence with electrochemiluminescence (ECL) reagent in the BioSpectrum 600 Imaging System (UVP, CA, USA).

Plasmids construction and cell transfection

To construct the overexpression plasmid, human circNUDT21 was amplified by PCR and cloned into the GV486 vector (CMV-left circular frame-MCS-right circular frame-EF1a-copGFP-SV40-Neomycin) (GeneChem, Shanghai, China). Small hairpin RNAs (shRNAs) targeting circNUDT21 were designed and cloned into the GV102 vector (hU6-MCS-CMV-GFP-SV40-Neomycin) (GeneChem). Both overexpression and knockdown plasmids were transfected into BC cells with the Lipofectamine 2000 Transfection Reagent (Invitrogen) according to the manufacturer's instructions, and stable cell lines were selected with G418. To construct cell lines overexpressing or silencing miRNAs, we purchased miRNA mimics and inhibitors from RiboBio (Guangzhou, China) and transfected them into cells with the Lipofectamine RNAiMAX Transfection Reagent (Invitrogen).

RNase R treatment

For RNase R treatment, the total RNA of BC cells was extracted and divided into two equal parts. After being incubated with or without 3 U/μg RNase R, the RNAs were reversely transcribed and analyzed by quantitative real-time-PCR.

RNA-FISH

The specific RNA-FISH probes for circNUDT21 and miR-16-1-3p were designed and synthesized by RiboBio. For the RNA-FISH assay, BC cells were fixed with 4% paraformaldehyde and permeabilized in 0.5% Triton X-100. Subsequently, cells were hybridized with the specific circRNA or miRNA probes at 37°C overnight. Then, the cells were counterstained with DAPI. All fluorescence images were captured using a Nikon A1R-si Laser Scanning Confocal Microscope (Nikon, Japan).

Pull-down assay with biotinylated circRNA probe

Briefly, 1×10^7 BC cells were fixed with 1% paraformaldehyde and lysed with lysis buffer (50 mM Tris-HCl pH 7.0, 10 mM EDTA, 1% SDS supplemented with 200 U/mL of a RNase inhibitor solution, and a cocktail of proteases inhibitor at 5 μL/mL). After preserving 20 μL supernatant as input, the remainder was divided into 2 equal parts and hybridized with the biotin-labeled circNUTD21 probe and the corresponding control probe at room temperature for 4–6 h. Subsequently, all of the samples were incubated with the M-280 streptavidin magnetic beads (Invitrogen) at 4°C overnight. Then, the beads were washed, and the RNA complexes combined with the beads were isolated using the RNeasy Mini Kit (QIAGEN, Dusseldorf, Germany). Finally, quantitative real-time-PCR was performed to validate the prediction of potential miRNAs interacting with circNUDT21. The sequences of probes are listed in Table S1.

Pull-down assay with biotinylated miRNA probe

BC cells were transfected with biotin-labeled miRNA mimics or matched nonsense control. After 48 h, cells were lysed (20 mM Tris-HCl pH 7.5, 100 mM KCl, 5 mM $MgCl_2$, 0.3% IGEPAL CA-630 supplemented with 200 U/mL RNase inhibitor solution, and a cocktail of protease inhibitor 5 μL/mL) and 50 μL of each lysate were reserved as input. The remaining lysates were incubated with M-280 streptavidin magnetic beads (Invitrogen) at 4°C overnight. On day 2, the RNA bound to magnetic beads were extracted with the RNeasy Mini Kit (QIAGEN), and the quantitative real-time-PCR was performed to analyze the circRNA abundance of each sample. The sequences of probes are listed in Table S1.

Xenografted tumor model

The animal experiments in this project were approved by the ethics committee of Tongji Medical College of the Huazhong University of Science and Technology. All BALB/c-nu mice (male, 3–5 weeks of age), acquired from the Center of Experimental Animals of Tongji Medical College of Huazhong University of Science and Technology, were randomly divided into 4 groups (n = 5/group). For the tumor formation assay, 2×10^6 BC cells were injected subcutaneously into the right side of each mouse. After injection, tumor volumes were observed using an external caliper and recorded using the equation $(L \times W^2)/2$. On day 30, all of the mice were euthanized, and tumors were excised, weighed, photographed, and subjected to pathological examination. All of the fluorescent images of xenografts in nude mice were captured with the *In Vivo* Optical Imaging System (*In Vivo* FX PRO, Bruker Corporation, MA, USA).

EdU labeling

Cells were incubated with EdU reagent (RiboBio) for 2 h at 37°C and then treated with ApolloR reaction cocktail according to the manufacturer's instructions. Images were collected using fluorescence microscopy (Olympus, Japan).

CCK-8 assay

BC cells were seeded into a 96-well plate at a density of 3×10^3/well with 100 μL serum-containing medium. After the cells were attached,

Figure 5. miR-16-1-3p can reverse the positive effects of circNUDT21 on bladder cancer progression

(A) The proliferation curves of BC cells transfected with circNUDT21 or co-transfected with miR-16-1-3p. (B) Representative EdU fluorescence images of indicated BC cells. Scale bar, 100 μm. (C) Mobility of BC cells measured with the wound healing assay. Scale bar, 200 μm. (D) Representative images of transwell migration and invasion assays for BC cells transfected with circNUDT21 or co-transfected with miR-16-1-3p. Scale bar, 100 μm. Data are represented as means ± SDs of 3 independent experiments. *$p < 0.05$.

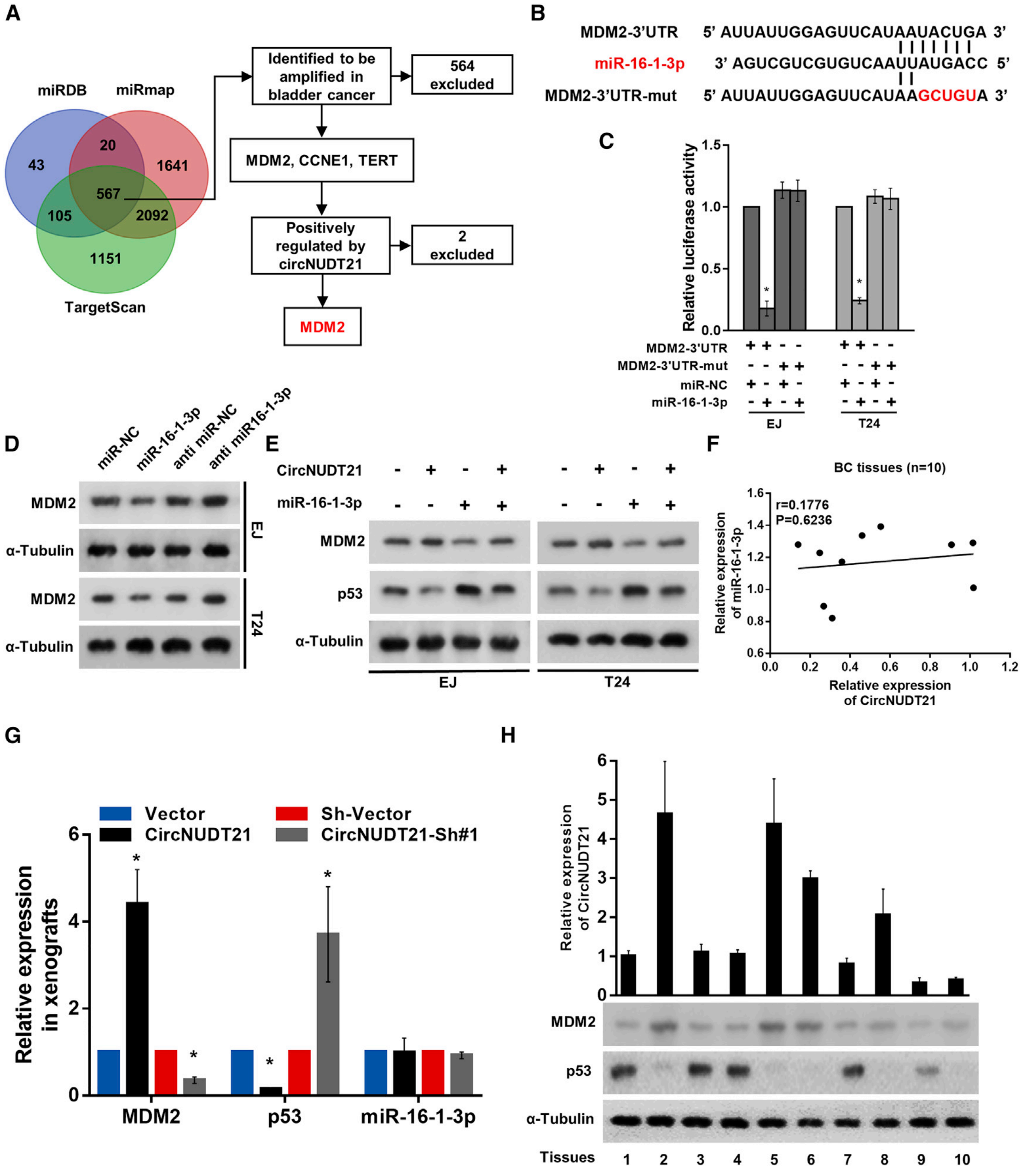

Figure 6. circNUDT21 sponges miR-16-1-3p to upregulate MDM2 expression in BC cells

(A) The Venn diagram showing the overlap of MDM2 in TargetScan, miRDB, and miRmap. (B) The target site between miR-16-1-3p and MDM2-3′ UTR. (C) Dual-luciferase reporter assay showing that miR-16-1-3p directly targeted MDM2-3′ UTR. (D) The protein levels of MDM2 detected by western blotting in miR-16-1-3p over-expressing and knockdown BC cells. (E) Western blotting assay showing the protein expression of MDM2 and p53 in indicated cells. (F) The expression correlation of circNUDT21 and miR-16-1-3p in BC tissues. (G) The expression levels of miR-16-1-3p, MDM2, and p53 in tumor xenografts from each group. (H) Western blotting and quantitative real-time-PCR assays showing the levels of circNUDT21, MDM2, and p53 in 10 BC tissue samples. Data are represented as means ± SDs of 3 independent experiments. *p < 0.05.

10 μL CCK-8 reagent (DOJINDO, Kumamoto, Japan) was added into wells and the plate was placed into an incubator for 1.5 h. Then, the optical density was measured at 450 nm using a microtiter plate reader.

Wound healing assay

BC cells were seeded into 6-well plates with complete medium. When the density of cells reaches ~90%, a 200-μL pipette tip was used to create a liner wound in the monolayer cells, and the medium was

replaced by serum-free medium. The distances between the opposite edges of the wound were measured using a microscope at 0 and 24 h after scratching.

Migration and invasion assays

The capacities of cell migration and invasion were evaluated using transwell chambers (Corning, NY, USA). For the migration assay, 4×10^4 cells suspended in 200 μL serum-free medium were seeded in the upper chamber of the transwell system, while the serum-containing medium was added into the lower chamber. For invasion assay, the transwell chambers were precoated with Matrigel. After incubation for 24 h, non-migrating or non-invasive cells remaining on the top surface were gently removed with a cotton swab. Cells migrated or invaded to the lower surface of the insert were fixed, stained, photographed, and counted under a light microscope.

Statistical analysis

Statistical analysis was carried out using the SPSS 19.0 software (SPSS, NY, USA). Differences between groups were analyzed by using the two-tailed Student's t test. $p < 0.05$ was considered statistically significant.

SUPPLEMENTAL INFORMATION

Supplemental information can be found online at https://doi.org/10.1016/j.omtn.2021.08.032.

ACKNOWLEDGMENTS

The study was supported by the National Natural Science Foundation of China (grant no. 81772714).

AUTHOR CONTRIBUTIONS

Conceptualization, T.H. and Y.X.; methodology & software, L.C.; validation & formal analysis, W.L. and Z.L.; writing – original draft, J.Z.; writing – review & editing, G.K. and L.L.; visualization, Z.C.; supervision, Y.X.; funding acquisition, T.H.

DECLARATION OF INTERESTS

The authors declare no competing interests.

REFERENCES

1. Antoni, S., Ferlay, J., Soerjomataram, I., Znaor, A., Jemal, A., and Bray, F. (2017). Bladder Cancer Incidence and Mortality: A Global Overview and Recent Trends. Eur. Urol. *71*, 96–108.
2. Cowan, R.A., McBain, C.A., Ryder, W.D., Wylie, J.P., Logue, J.P., Turner, S.L., Van der Voet, J., Collins, C.D., Khoo, V.S., and Read, G.R. (2004). Radiotherapy for muscle-invasive carcinoma of the bladder: results of a randomized trial comparing conventional whole bladder with dose-escalated partial bladder radiotherapy. Int. J. Radiat. Oncol. Biol. Phys. *59*, 197–207.
3. Abufaraj, M., Dalbagni, G., Daneshmand, S., Horenblas, S., Kamat, A.M., Kanzaki, R., Zlotta, A.R., and Shariat, S.F. (2018). The Role of Surgery in Metastatic Bladder Cancer: A Systematic Review. Eur. Urol. *73*, 543–557.
4. Memczak, S., Jens, M., Elefsinioti, A., Torti, F., Krueger, J., Rybak, A., Maier, L., Mackowiak, S.D., Gregersen, L.H., Munschauer, M., et al. (2013). Circular RNAs are a large class of animal RNAs with regulatory potency. Nature *495*, 333–338.
5. Ju, H.Q., Zhao, Q., Wang, F., Lan, P., Wang, Z., Zuo, Z.X., Wu, Q.N., Fan, X.J., Mo, H.Y., Chen, L., et al. (2019). A circRNA signature predicts postoperative recurrence in stage II/III colon cancer. EMBO Mol. Med. *11*, e10168.
6. Ng, W.L., Mohd Mohidin, T.B., and Shukla, K. (2018). Functional role of circular RNAs in cancer development and progression. RNA Biol. *15*, 995–1005.
7. Li, S., and Han, L. (2019). Circular RNAs as promising biomarkers in cancer: detection, function, and beyond. Genome Med. *11*, 15.
8. Wang, Y., Yin, L., and Sun, X. (2020). CircRNA hsa_circ_0002577 accelerates endometrial cancer progression through activating IGF1R/PI3K/Akt pathway. J. Exp. Clin. Cancer Res. *39*, 169.
9. Xia, B., Zhao, Z., Wu, Y., Wang, Y., Zhao, Y., and Wang, J. (2020). Circular RNA circTNPO3 Regulates Paclitaxel Resistance of Ovarian Cancer Cells by miR-1299/NEK2 Signaling Pathway. Mol. Ther. Nucleic Acids *21*, 780–791.
10. Li, J., Ma, M., Yang, X., Zhang, M., Luo, J., Zhou, H., Huang, N., Xiao, F., Lai, B., Lv, W., and Zhang, N. (2020). Circular HER2 RNA positive triple negative breast cancer is sensitive to Pertuzumab. Mol. Cancer *19*, 142.
11. Zhang, H., Sheng, C., Yin, Y., Wen, S., Yang, G., Cheng, Z., and Zhu, Q. (2015). PABPC1 interacts with AGO2 and is responsible for the microRNA mediated gene silencing in high grade hepatocellular carcinoma. Cancer Lett. *367*, 49–57.
12. Xiong, M., Chen, L., Zhou, L., Ding, Y., Kazobinka, G., Chen, Z., and Hou, T. (2019). NUDT21 inhibits bladder cancer progression through ANXA2 and LIMK2 by alternative polyadenylation. Theranostics *9*, 7156–7167.
13. Schmitz-Dräger, B.J., Kushima, M., Goebell, P., Jax, T.W., Gerharz, C.D., Bültel, H., Schulz, W.A., Ebert, T., and Ackermann, R. (1997). p53 and MDM2 in the development and progression of bladder cancer. Eur. Urol. *32*, 487–493.
14. Hosono, Y., Niknafs, Y.S., Prensner, J.R., Iyer, M.K., Dhanasekaran, S.M., Mehra, R., Pitchiaya, S., Tien, J., Escara-Wilke, J., Poliakov, A., et al. (2017). Oncogenic Role of THOR, a Conserved Cancer/Testis Long Non-coding RNA. Cell *171*, 1559–1572.e20.
15. Spizzo, R., Almeida, M.I., Colombatti, A., and Calin, G.A. (2012). Long non-coding RNAs and cancer: a new frontier of translational research? Oncogene *31*, 4577–4587.
16. Jin, M., Lu, S., Wu, Y., Yang, C., Shi, C., Wang, Y., and Huang, G. (2020). Hsa_circ_0001944 promotes the growth and metastasis in bladder cancer cells by acting as a competitive endogenous RNA for miR-548. J. Exp. Clin. Cancer Res. *39*, 186.
17. Su, Y., Feng, W., Shi, J., Chen, L., Huang, J., and Lin, T. (2020). circRIP2 accelerates bladder cancer progression via miR-1305/Tgf-β2/smad3 pathway. Mol. Cancer *19*, 23.
18. Feng, Q.Q., Dong, Z.Q., Zhou, Y., Zhang, H., and Long, C. (2018). miR-16-1-3p targets TWIST1 to inhibit cell proliferation and invasion in NSCLC. Bratisl. Lek Listy *119*, 60–65.
19. Wang, T., Hou, J., Li, Z., Zheng, Z., Wei, J., Song, D., Hu, T., Wu, Q., Yang, J.Y., and Cai, J.C. (2017). miR-15a-3p and miR-16-1-3p Negatively Regulate Twist1 to Repress Gastric Cancer Cell Invasion and Metastasis. Int. J. Biol. Sci. *13*, 122–134.
20. Ye, T., Liang, Y., Zhang, D., and Zhang, X. (2020). MicroRNA-16-1-3p Represses Breast Tumor Growth and Metastasis by Inhibiting PGK1-Mediated Warburg Effect. Front. Cell Dev. Biol. *8*, 615154.
21. Guarnerio, J., Zhang, Y., Cheloni, G., Panella, R., Mae Katon, J., Simpson, M., Matsumoto, A., Papa, A., Loretelli, C., Petri, A., et al. (2019). Intragenic antagonistic roles of protein and circRNA in tumorigenesis. Cell Res. *29*, 628–640.
22. Liang, D., Tatomer, D.C., Luo, Z., Wu, H., Yang, L., Chen, L.L., Cherry, S., and Wilusz, J.E. (2017). The Output of Protein-Coding Genes Shifts to Circular RNAs When the Pre-mRNA Processing Machinery Is Limiting. Mol. Cell *68*, 940–954.e3.
23. Konopleva, M., Martinelli, G., Daver, N., Papayannidis, C., Wei, A., Higgins, B., Ott, M., Mascarenhas, J., and Andreeff, M. (2020). MDM2 inhibition: an important step forward in cancer therapy. Leukemia *34*, 2858–2874.
24. Wu, D., Niu, X., Tao, J., Li, P., Lu, Q., Xu, A., Chen, W., and Wang, Z. (2017). MicroRNA-379-5p plays a tumor-suppressive role in human bladder cancer growth and metastasis by directly targeting MDM2. Oncol. Rep. *37*, 3502–3508.
25. Oliner, J.D., Saiki, A.Y., and Caenepeel, S. (2016). The Role of MDM2 Amplification and Overexpression in Tumorigenesis. Cold Spring Harb. Perspect. Med. *6*, a026336.

26. Finlay, C.A. (1993). The mdm-2 oncogene can overcome wild-type p53 suppression of transformed cell growth. Mol. Cell. Biol. *13*, 301–306.

27. Jones, S.N., Hancock, A.R., Vogel, H., Donehower, L.A., and Bradley, A. (1998). Overexpression of Mdm2 in mice reveals a p53-independent role for Mdm2 in tumorigenesis. Proc. Natl. Acad. Sci. USA *95*, 15608–15612.

28. Ciccarese, C., Massari, F., Blanca, A., Tortora, G., Montironi, R., Cheng, L., Scarpelli, M., Raspollini, M.R., Vau, N., Fonseca, J., and Lopez-Beltran, A. (2017). Tp53 and its potential therapeutic role as a target in bladder cancer. Expert Opin. Ther. Targets *21*, 401–414.

29. Garczyk, S., Ortiz-Brüchle, N., Schneider, U., Lurje, I., Guricova, K., Gaisa, N.T., Lorsy, E., Lindemann-Docter, K., Heidenreich, A., and Knüchel, R. (2020). Next-Generation Sequencing Reveals Potential Predictive Biomarkers and Targets of Therapy for Urothelial Carcinoma in Situ of the Urinary Bladder. Am. J. Pathol. *190*, 323–332.

30. Kim, J.Y., Lee, R., Xiao, G., Forbes, D., and Bargonetti, J. (2020). MDM2-C Functions as an E3 Ubiquitin Ligase. Cancer Manag. Res. *12*, 7715–7724.

31. Chen, L., Yang, X., Zhao, J., Xiong, M., Almaraihah, R., Chen, Z., and Hou, T. (2020). Circ_0008532 promotes bladder cancer progression by regulation of the miR-155-5p/miR-330-5p/MTGR1 axis. J. Exp. Clin. Cancer Res. *39*, 94.

OMTN, Volume *26*

Supplemental information

circNUDT21 promotes bladder cancer progression

by modulating the miR-16-1-3p/MDM2/p53 axis

Liang Chen, Wencheng Li, Zhiqin Li, Yarong Song, Jun Zhao, Zhaohui Chen, Gallina Kazobinka, Lulu Li, Yifei Xing, and Teng Hou

Supplemental Table 1. The sequences of primers, oligonucleotides and probes used in this study.

**PCR**	CircNUDT21-divergent	Forward	AATGCACCAGGATATGGACCC
		Reverse	TGAAGCTGAACCAGAAACAACT
	CircNUDT21-convergent	Forward	CCTTGTTTGCAGTCCCTAAAAAT
		Reverse	CAACAGCTGAGGGAGACTAGAA
	GAPDH-divergent	Forward	TCCCCCACCACACTGAATCT
		Reverse	ACCACCTGGTGCTCAGTGTA
	GAPDH-convergent	Forward	TTTGGCTACAGCAACAGGGTG
		Reverse	TACATGGCAACTGTGAGGAGGG
	Circ_0039441-divergent	Forward	GTTTGGTTTTGAGAACTTTTCTGGT
		Reverse	GGGTCCATATCCTGGTGCAT
	Circ_0039442-divergent	Forward	GTTTGGTTTTGAGAACTTTTCTGGT
		Reverse	GGCGTAGGCACGCCG
**qRT-PCR**	CircNUDT21	Forward	AATGCACCAGGATATGGACCC
		Reverse	TGAAGCTGAACCAGAAACAACT
	NUDT21	Forward	CCCCACCTTAGGATGGTGAA
		Reverse	GGAGTTGCACCTTGGACTCA
	MDM2	Forward	ACGACAAAGAAAACGCCACA
		Reverse	GTAACTTGATATACACCAGCATCAA
	GAPDH	Forward	AAATCAAGTGGGGCGATGCTG
		Reverse	GCAGGAGGCATTGCTGATGAT
	miR-3162-5p	Forward	CGCGTTAGGGAGTAGAAGGGT
		Reverse	AGTGCAGGGTCCGAGGTATT

miR-6511b-5p	Forward	CTGCAGGCAGAAGTGGGG
	Reverse	AGTGCAGGGTCCGAGGTATT
miR-4793-5p	Forward	CGACATCCTGCTCCACAGGG
	Reverse	AGTGCAGGGTCCGAGGTATT
miR-6129	Forward	CGCGTGAGGGAGTTGGG
	Reverse	AGTGCAGGGTCCGAGGTATT
miR-4739	Forward	GGGAGGAGGAGCGGAGG
	Reverse	AGTGCAGGGTCCGAGGTATT
miR-16-1-3p	Forward	CGCGCCAGTATTAACTGTGC
	Reverse	AGTGCAGGGTCCGAGGTATT
miR-4482-5p	Forward	GCGCGAATTGAATAGTGAGCA
	Reverse	AGTGCAGGGTCCGAGGTATT
miR-8052	Forward	CGCGGGACTGTAGAGGGC
	Reverse	AGTGCAGGGTCCGAGGTATT
miR-4635	Forward	GCGCGTCTTGAAGTCAGAAC
	Reverse	AGTGCAGGGTCCGAGGTATT
miR-6874-5p	Forward	CGATGGAGCTGGAACCAGA
	Reverse	AGTGCAGGGTCCGAGGTATT
miR-19b-1-5p	Forward	GCGAGTTTTGCAGGTTTGCA
	Reverse	AGTGCAGGGTCCGAGGTATT
miR-3130-5p	Forward	GCGACCCAGTCTCCGGTG
	Reverse	AGTGCAGGGTCCGAGGTATT
miR-6834-5p	Forward	GCGGTGAGGGACTGGGAT

	Reverse	AGTGCAGGGTCCGAGGTATT
miR-6794-5p	Forward	CGCAGGGGGACTGGGG
	Reverse	AGTGCAGGGTCCGAGGTATT
miR-6760-5p	Forward	CGCAGGGAGAAGGTGGAAG
	Reverse	AGTGCAGGGTCCGAGGTATT
miR-509-5p	Forward	CGCGTACTGCAGACAGTGG
	Reverse	AGTGCAGGGTCCGAGGTATT
miR-3911	Forward	GCGTGTGTGGATCCTGGAG
	Reverse	AGTGCAGGGTCCGAGGTATT
miR-4686	Forward	GCGTATCTGCTGGGCTTTCT
	Reverse	AGTGCAGGGTCCGAGGTATT
miR-6825-5p	Forward	CGTGGGGAGGTGTGGAGT
	Reverse	AGTGCAGGGTCCGAGGTATT
miR-5047	Forward	CGTTGCAGCTGCGGTTG
	Reverse	AGTGCAGGGTCCGAGGTATT
miR-6829-5p	Forward	GCGTGGGCTGCTGAGAA
	Reverse	AGTGCAGGGTCCGAGGTATT
miR-6127	Forward	CGCGTGAGGGAGTGGGT
	Reverse	AGTGCAGGGTCCGAGGTATT
miR-6133	Forward	CGCGTGAGGGAGGAGGT
	Reverse	AGTGCAGGGTCCGAGGTATT
miR-4510	Forward	CGCGTGAGGGAGTAGGATGT
	Reverse	AGTGCAGGGTCCGAGGTATT

	miR-22-3p	Forward	GCGAAGCTGCCAGTTGAAG
		Reverse	AGTGCAGGGTCCGAGGTATT
	miR-3199	Forward	GCGAGGGACTGCCTTAGGAG
		Reverse	AGTGCAGGGTCCGAGGTATT
	miR-1301-3p	Forward	TTGCAGCTGCCTGGGAGT
		Reverse	AGTGCAGGGTCCGAGGTATT
	U6	Forward	CTCGCTTCGGCAGCACA
		Reverse	AACGCTTCACGAATTTGCGT
**RT-PCR**	miR-3162-5p	GTCGTATCCAGTGCAGGGTCCGAGGTATTCGCACTGGATACGACCTCCCC	
	miR-6511b-5p	GTCGTATCCAGTGCAGGGTCCGAGGTATTCGCACTGGATACGACTGTCAG	
	miR-4793-5p	GTCGTATCCAGTGCAGGGTCCGAGGTATTCGCACTGGATACGACCCTCTG	
	miR-6129	GTCGTATCCAGTGCAGGGTCCGAGGTATTCGCACTGGATACGACTATACA	
	miR-4739	GTCGTATCCAGTGCAGGGTCCGAGGTATTCGCACTGGATACGACAGGGCC	
	miR-16-1-3p	GTCGTATCCAGTGCAGGGTCCGAGGTATTCGCACTGGATACGACTCAGCA	
	miR-4482-5p	GTCGTATCCAGTGCAGGGTCCGAGGTATTCGCACTGGATACGACCTGGGT	
	miR-8052	GTCGTATCCAGTGCAGGGTCCGAGGTATTCGCACTGGATACGACGCTCAT	
	miR-4635	GTCGTATCCAGTGCAGGGTCCGAGGTATTCGCACTGGATACGACTTGCGG	
	miR-6874-5p	GTCGTATCCAGTGCAGGGTCCGAGGTATTCGCACTGGATACGACGCCTGA	
	miR-19b-1-5p	GTCGTATCCAGTGCAGGGTCCGAGGTATTCGCACTGGATACGACGCTGGA	

miR-3130-5p	GTCGTATCCAGTGCAGGGTCCGAGGTATTCGCACTGGATACGACAGGCTG
miR-6834-5p	GTCGTATCCAGTGCAGGGTCCGAGGTATTCGCACTGGATACGACCCACAA
miR-6794-5p	GTCGTATCCAGTGCAGGGTCCGAGGTATTCGCACTGGATACGACGCTCAC
miR-6760-5p	GTCGTATCCAGTGCAGGGTCCGAGGTATTCGCACTGGATACGACTCTGCA
miR-509-5p	GTCGTATCCAGTGCAGGGTCCGAGGTATTCGCACTGGATACGACTGATTG
miR-3911	GTCGTATCCAGTGCAGGGTCCGAGGTATTCGCACTGGATACGACTGCCTC
miR-4686	GTCGTATCCAGTGCAGGGTCCGAGGTATTCGCACTGGATACGACAACACC
miR-6825-5p	GTCGTATCCAGTGCAGGGTCCGAGGTATTCGCACTGGATACGACATGCTG
miR-5047	GTCGTATCCAGTGCAGGGTCCGAGGTATTCGCACTGGATACGACACCTTA
miR-6829-5p	GTCGTATCCAGTGCAGGGTCCGAGGTATTCGCACTGGATACGACTGCCCC
miR-6127	GTCGTATCCAGTGCAGGGTCCGAGGTATTCGCACTGGATACGACCCTCCC
miR-6133	GTCGTATCCAGTGCAGGGTCCGAGGTATTCGCACTGGATACGACTACCCA
miR-4510	GTCGTATCCAGTGCAGGGTCCGAGGTATTCGCACTGGATACGACAACCAT
miR-22-3p	GTCGTATCCAGTGCAGGGTCCGAGGTATTCGCACTGGATACGACACAGTT
miR-3199	GTCGTATCCAGTGCAGGGTCCGAGGTATTCGCACTGGATACGACAACTTT
miR-1301-3p	GTCGTATCCAGTGCAGGGTCCGAGGTATTCGCACTGGATACGACGAAGTC
U6	AACGCTTCACGAATTTGCGT

**RNA pull-down**	CircNUDT21-probe	TCCTGACGACCCAGTATCTGCTCAACAGCTGAGG-Biotin
	Oligo probe	CCTCAGCTGTTGAGCAGATACTGGGTCGTCAGGA-Biotin
	Bio-miR-16-1-3p	CCAGUAUUAACUGUGCUGCUGA-Biotin
	Bio-miR-16-1-3p-mut	AACUGCGUAAAGGUGAUAUGAC-Biotin
